# Anti‐inflammatory actions of acetate, propionate, and butyrate in fetal mouse jejunum cultures ex vivo and immature small intestinal cells in vitro

**DOI:** 10.1002/fsn3.2682

**Published:** 2022-01-18

**Authors:** Shengnan Huang, Yanan Gao, Ziwei Wang, Xue Yang, Jiaqi Wang, Nan Zheng

**Affiliations:** ^1^ Key Laboratory of Quality & Safety Control for Milk and Dairy Products of Ministry of Agriculture and Rural Affairs Institute of Animal Sciences Chinese Academy of Agricultural Sciences Beijing China; ^2^ Laboratory of Quality and Safety Risk Assessment for Dairy Products of Ministry of Agriculture and Rural Affairs Institute of Animal Sciences Chinese Academy of Agricultural Sciences Beijing China; ^3^ Milk and Dairy Product Inspection Center of Ministry of Agriculture and Rural Affairs Institute of Animal Sciences Chinese Academy of Agricultural Sciences Beijing China; ^4^ State Key Laboratory of Animal Nutrition Institute of Animal Sciences Chinese Academy of Agricultural Sciences Beijing China; ^5^ College of Food Science and Engineering Qingdao Agricultural University Qingdao China

**Keywords:** inflammation of immature small intestine, inflammatory cytokines, RNA‐seq, short‐chain fatty acids, signaling pathway

## Abstract

Necrotizing enterocolitis (NEC) is an intestinal disease that frequently occurs in premature infants. Presently, there is no effective therapy for NEC. Therefore, the key to reduce the incidence rate of NEC is to take effective intervention measures as early as possible. Short‐chain fatty acids (SCFAs) (acetate, propionate, and butyrate), the principal terminal products of enterobacteria fermentation, play anti‐inflammatory actions in mature intestinal cells. However, few studies focus on their roles in immature intestine. Here, we evaluated the anti‐inflammatory actions of SCFAs ex vivo with ICR fetal mouse jejunum cultures and explored the potential anti‐inflammatory regulators through RNA‐seq and then verified them in vitro with human fetal small intestinal epithelial FHs 74 Int cells. In this study, we found that acetate, propionate, and butyrate decreased IL‐1β‐induced production of CXCL2 ex vivo and IL‐8 and IL‐6 in vitro significantly (*p* < .05). Furthermore, the inhibitors of NF‐κB p65, JNK1/2, and ERK1/2 pathways, which were selected from RNA‐seq and depressed by SCFAs, also significantly decreased IL‐8 and IL‐6 productions induced by IL‐1β (*p* < .05). Therefore, our results showed that acetate, propionate, and butyrate ameliorated the fetal small intestine inflammatory response induced by IL‐1β through inhibiting ERK1/2 pathway; NF‐κB p65, JNK1/2, and ERK1/2 pathways; or NF‐κB p65 and ERK1/2 pathways, respectively. These findings suggested that SCFAs may be a new therapy agent for NEC.

## Graphical abstract

In this study, acetate, propionate, and butyrate had a protective effect on fetal ICR mouse jejunum culture inflammation and human fetal small intestinal epithelial FHs Int 74 cell inflammation induced by IL‐1β through inhibiting ERK1/2 pathway; NF‐κB p65, JNK1/2, and ERK1/2 pathways; or NF‐κB p65 and ERK1/2 pathways, respectively.

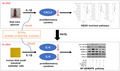

## 1 INTRODUCTION

Necrotizing enterocolitis (NEC) is a prevalent inflammatory bowel disease in newborns, which mainly occurs in preterm infants (Nanthakumar et al., 2011). The incidence of NEC among preterm infants in Australia, Canada, and Italy was 7%–9%; what's more, the mortality rate is high from NEC (10%–50%) (Alganabi et al., 2019). The risk of NEC is in inverse proportion to gestational age and birthweight (Zani & Pierro, 2015). Although the pathophysiology of NEC is complex, three major risk factors have been consistently described: prematurity, abnormal bacterial colonization, and formula feeding (Alganabi et al., 2019; Jung et al., 2017; Niño et al., 2016). Preterm infants are more likely to develop NEC because of reduced intestinal barrier function, damaged intestinal immune system, and increased inflammatory tendency compared with full‐term infants (Denning & Prince, 2018). So far, there is no specific treatment for NEC due to the multifactorial nature of its pathogenesis. The key to reduce the incidence of NEC is to take effective intervention measures early.

Growing evidence demonstrates that the intestinal flora is a key factor in maintaining intestinal homeostasis, which can be achieved in part through the release of short‐chain fatty acids (SCFAs), the main metabolites of indigestible sugars fermented by intestinal bacteria (Tan et al., 2014). The concentrations of SCFAs in colon in human range from 50 to 150 mM (Wu et al., 2017). Among the total SCFAs, acetate, propionate, and butyrate are the main types with the ratio of 60:25:15 (Li et al., 2018), accounted for more than 95% (Sun et al., 2016). SCFAs have been shown to promote intestinal integrity (Burger‐van et al., 2009; Fellows et al., 2018; Ferreira et al., 2012; Vinolo et al., 2011), regulate metabolic disorders, and modulate immune responses (Bhutia & Ganapathy, 2015; Nastasi et al., 2015). At present, studies have demonstrated that SCFAs also inhibit inflammatory reactions in mice colon (Chen et al., 2018; Hung & Suzuki, 2018; Kim et al., 2018; Singh et al., 2014) or human colonic cell line (such as Caco‐2) (Hung & Suzuki, 2018). Furthermore, proinflammatory cytokine signaling activates NF‐κB and MAPK, the main inflammatory signaling pathways in intestinal inflammation, and these signaling pathways are important targets for treatment (Tedelind et al., 2007). Previous studies have reported that SCFAs ameliorate inflammation through inhibiting NF‐κB and MAPK pathways (Chen et al., 2018; Kobayashi et al., 2017; Tedelind et al., 2007). In addition, SCFAs also reduced intestinal inflammation via inhibition of histone deacetylase (HDAC) (Chang et al., 2014; Furusawa et al., 2013). However, in the current studies, the anti‐inflammatory actions of SCFAs on intestinal inflammation are mostly focused on the large intestine using mouse colon or human colonic cell line, rather than the small intestine, especially the immature small intestine.

FHs 74 Int cells originated from small intestine of normal human fetal are highly consistent with jejunum of fetal mouse, and they are suitable models for the immature fetal small intestine. Therefore, in this study, we used ICR fetal mouse jejunum cultures model ex vivo and human fetal intestinal epithelial FHs 74 Int cells model in vitro of inflammation to simulate NEC in preterm infants. Transcriptomics allows an extensive analysis of genes in an organism to explore their functions (Song et al., 2017; Wang et al., 2019). RNA‐seq is an ordinary technique for transcription, which has the advantages of low background signal, high resolution, and good repeatability (Jung et al., 2017; Wang et al., 2009). It has been prevalently used to reveal the molecular mechanism of diseases and specific biological processes. The objective of this study was to demonstrate the anti‐inflammatory actions of SCFAs on immature small intestinal inflammation. RNA‐seq was applied to evaluate the gene expression changes and distribution in fetal mouse jejunum models and then verified by the human fetal small intestinal cell inflammation models to determine the molecular mechanism of this process. This could provide a new treatment to prevent the development of NEC in preterm infants.

## 2 MATERIALS AND METHODS

### 2.1 Animal model and treatments

Pregnant ICR mice were raised in specific pathogen‐free environment and fed food and water freely. Fetal mice were removed by cesarean delivery at E18.5 (Zheng et al., 2020). The jejunum was obtained from fetal mouse and then cut into 3‐mm segments and preserved in organ culture medium at 37°C as previously mentioned (Zheng et al., 2020). This medium was prepared by Opti‐MEM I medium supplemented with 10% FBS, 2 mM L‐glutamine, 10 mM HEPES buffer (Gibco), 200 ng/ml epidermal growth factor (EGF) from mouse, ITS liquid media supplement (100×) (Sigma), 200 nM hydrocortisone (MCE), and penicillin–streptomycin–amphotericin B solution (100×) (Solarbio). After 2 h, jejunum tissues were treated with or without SCFAs (acetate, propionate, and butyrate) (Sigma) for 1 h and then cultured with mouse IL‐1β (R&D Systems) for 24 h. Finally, the supernatants of jejunum cultures were collected and stored at −20°C for ELISA assay. The tissues were collected and stored at −80°C for RNA extraction and transcriptome analysis. Animal experiments were approved by Animal Experimental Ethics Committee of Institute of Animal Sciences, Chinese Academy of Agricultural Science (IAS2019‐65).

### 2.2 Cell culture

FHs 74 Int cells, human fetal small intestinal epithelial cell line, were purchased from American Type Culture Collection (ATCC). FHs 74 Int cells were cultured in Hybri­Care Medium (ATCC) supplemented with 10% FBS, antibiotics (100 U/ml penicillin, 0.1 mg/ml streptomycin) (Beyotime, Shanghai, China), and 30 ng/ml human EGF (Sigma). The cells were cultured in an incubator containing 5% CO_2_ at 37°C, and medium was changed every 2–3 days.

### 2.3 ELISA

The concentrations of proinflammatory cytokines CXCL2 (murine homologue of IL‐8), IL‐8, and IL‐6 (human) in tissue or cell culture media were assayed by ELISA kit (R&D Systems; Abcam, Cambridge, MH) according to instructions for use. The absorbance of the sample was detected by microplate reader at 450 nm (Thermo Scientific).

### 2.4 RNA‐seq and data analysis

Total RNA from fetal mouse jejunum tissues was extracted with Trizol (Invitrogen) based on instructions for use. RNA quality was checked using Agilent 2100 Bioanalyzer (Agilent Technologies) and agarose gel electrophoresis. After total RNA was extracted, eukaryotic mRNA was enriched by Oligo (dT) beads. Then, fragmentation buffer was used to break the enriched mRNA into short fragments. First strand of cDNA was synthesized in reverse transcriptase system using fragmentized mRNA as template and random oligonucleotide as primer. Subsequently, RNase H was used to degrade the RNA strand, and second strand of cDNA was synthesized using dNTPs as raw materials in the DNA polymerase I system. Then, the cDNA fragments were purified, end‐repaired, a‐tailed, and ligated to Illumina sequencing adapters. Agarose gel electrophoresis was used to select ligation product size, and PCR amplification was performed. Finally, sequencing was performed using Illumina HiSeq2500 by Gene Denovo Biotechnology Co., Ltd. Raw data obtained from sequencing were filtered to get clean data. The clean data were then mapped to mouse reference genome. Gene expression profiles were used to perform principal component analysis (PCA). Differential expression genes (DEGs) were identified using DESeq2 package, taking absolute fold change ≥2 and false discovery rate (FDR) <0.05 as a threshold. Finally, KEGG enrichment analysis of DEGs was carried out using R based on the hypergeometric test. KEGG pathway with *p* < .05 was considered to be significantly enriched in DEGs.

### 2.5 qRT‐PCR analysis

Total RNA was isolated with Trizol from fetal mouse jejunum tissues and FHs 74 Int cells, and RNA was reverse‐transcribed into cDNA by PrimeScript RT reagent Kit (TaKaRa). Then, TB Green Premix Ex Taq Ⅱ (TaKaRa) was used for real‐time PCR of cDNA. To ensure the reliability of the RNA‐seq results, ten DEGs were chosen and detected by qRT‐PCR. These ten genes were randomly selected from the DEGs between the propionate+IL‐1β and IL‐1β groups. The primers of proinflammatory cytokines and ten DEGs are shown in Table S1. β‐Actin gene was chosen as an internal reference gene to standardize the expression data. The relative mRNA expression was calculated by 2^‐∆∆Ct^ method.

### 2.6 Western blot analysis

Differentially expressed pathways were verified by Western blot. Protein extraction and Western blot were performed as previously mentioned (Yang et al., 2019). Antibodies were used according to the manufacturer's instruction: NF‐κB p65, pNF‐κB p65, ERK, pERK1/2, JNK (Cell Signaling Technology), pJNK1/2 (Invitrogen), and goat anti‐rabbit IgG/HRP (Bioss). The bands were visualized with enhanced chemiluminescence (ECL) (Thermo Scientific) on Tanon‐5200 Chemiluminescence Imaging System (Tanon). ImageJ 2 x software was used to quantify the band densities.

### 2.7 Inhibition assay

FHs 74 Int cells were pretreated with BAY 11–7085 (NF‐κB p65 inhibitor, 10 mM; MCE), SP600125 (JNK1/2 inhibitor, 10 mM; MCE), or U0126 (ERK1/2 inhibitor, 20 mM; MCE) in the presence or absence for 1 h before IL‐1β treatment for 24 h. The mRNA levels of IL‐8 and IL‐6 were evaluated by qRT‐PCR.

### 2.8 Statistical analysis

All data are analyzed with GraphPad Prism 5 and shown as mean ± *SD* of three independent experiments with at least three replicates per independent experiments. The means of two groups were compared by unpaired Student's *t* test, and the means of multiple groups were compared by the one‐way ANOVA and the Tukey post hoc test. *p* < .05 was judged a significant difference.

## 3 RESULTS

### 3.1 SCFAs inhibited CXCL2 expression in IL‐1β‐induced fetal mouse jejunum organ cultures

**FIGURE 1:**
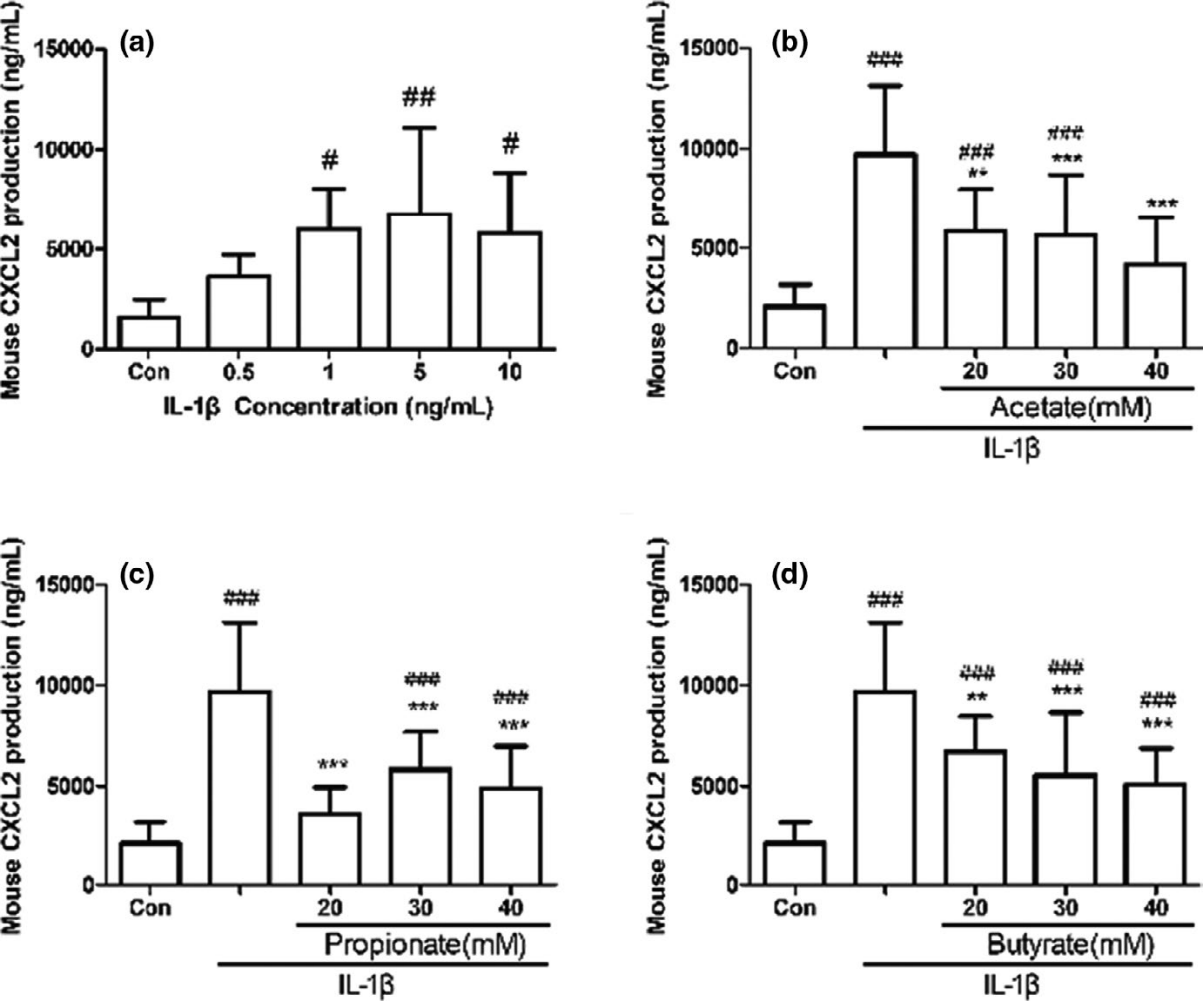
Sensitivity of CXCL2 expression level in fetal mouse jejunum cultures to IL‐1β and SCFAs. Fetal mouse jejunum cultures were stimulated with or without IL‐1β (0.5–10 ng/ml) for 24 h (a). Fetal mouse jejunum cultures were pretreated with or without 20−40 mM acetate (b), propionate (c), or butyrate (d) for 1 h, and then cultured with IL‐1β (1 ng/ml) for 24 h. CXCL2 concentration in fetal mouse jejunum cultures supernatant was measured by ELISA kit. Data are represented as mean ± *SD*. ∗∗*p* < .01, ∗∗∗*p* < .001 vs. the IL‐1β group. #*p* < .05, ##*p* < .01 vs. the control group

In order to construct the fetal mouse jejunal model of inflammation, we treated fetal mouse jejunal organ cultures with different IL‐1β concentrations (0.5–10 ng/ml). As shown in Figure 1a, CXCL2 productions were significantly increased in fetal mouse jejunal organ cultures treated with 1–10 ng/ml IL‐1β (*p* < .05). Accordingly, 1 ng/ml was selected as the concentration of IL‐1β to construct the fetal mouse jejunal model of inflammation. Treatment with acetate, propionate, or butyrate at all test concentrations (20–40 mM) significantly reduced the increase in CXCL2 secretion induced by IL‐1β (Figure 1b,c,d, *p* < .05). 30 and 40 mM of SCFAs had the best inhibitory effect, and there was no significant difference. Therefore, 30 mM of SCFAs was used in fetal mouse jejunal model in the following RNA‐seq analysis.

### 3.2 PCA

**FIGURE 2:**
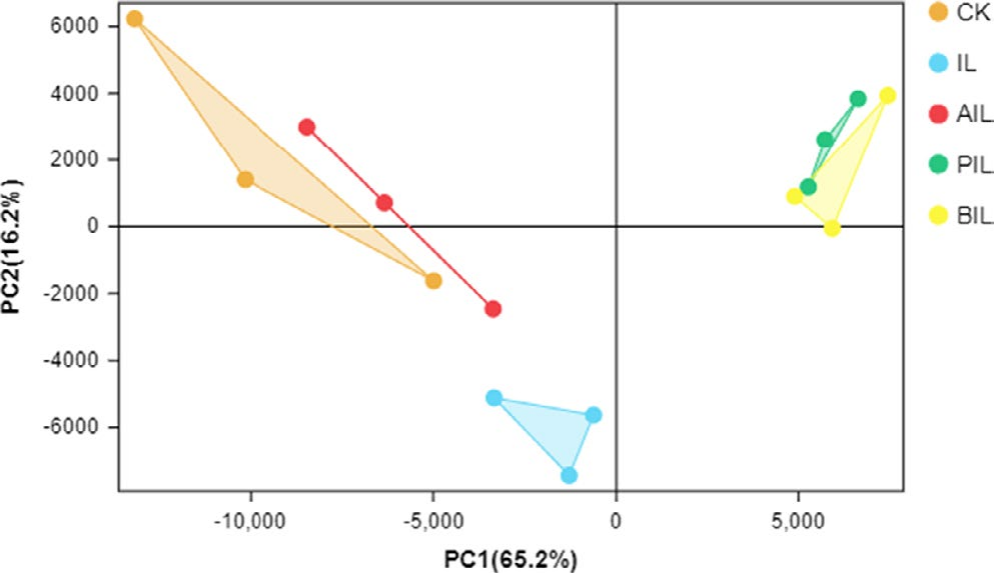
Principal component analysis (PCA) in the CK, IL, AIL, PIL, and BIL groups. CK, control group. IL, IL‐1β group. AIL, acetate+IL‐1β group. PIL, propionate+IL‐1β group. BIL, butyrate+IL‐1β group

Principal component analysis was performed on three biological replicate samples from the control, IL‐1β, acetate+IL‐1β, propionate+IL‐1β, and butyrate+IL‐1β groups. As seen in Figure 2, the samples in each treatment group had good repeatability, and the samples of five treatment groups were separated well.

### 3.3 KEGG enrichment analysis of DEGs

**FIGURE 3:**
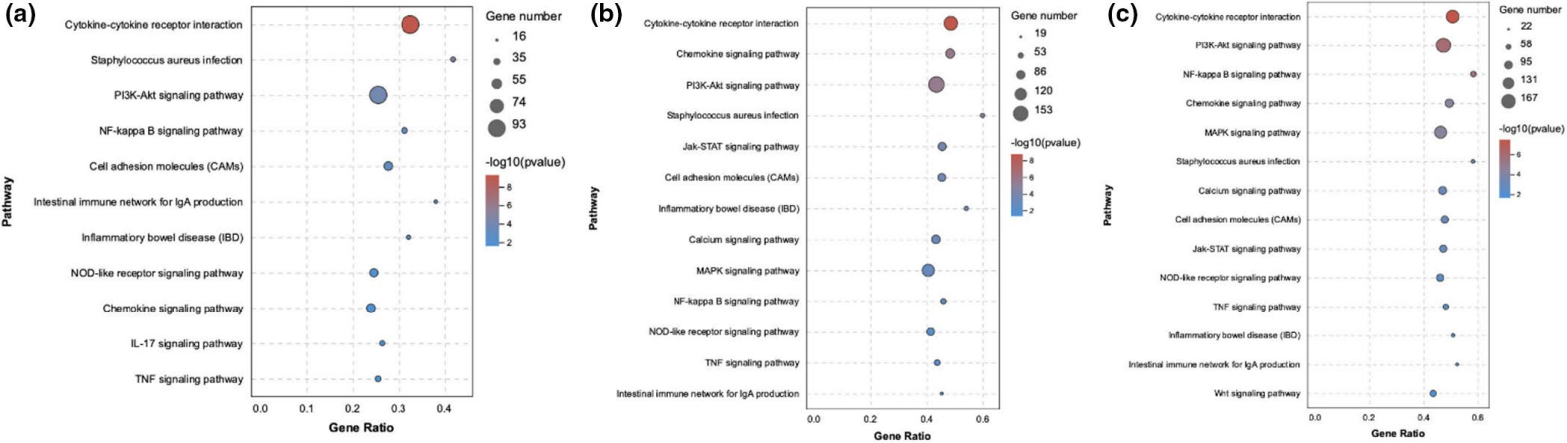
Significantly enriched KEGG pathways related to inflammation for DEGs in the IL vs. AIL group (a), IL vs. PIL group (b), IL vs. BIL group (c). IL, IL‐1β group. AIL, acetate+IL‐1β group. PIL, propionate+IL‐1β group. BIL, butyrate+IL‐1β group

KEGG enrichment analysis of DEGs between the acetate+IL‐1β and IL‐1β groups revealed 90 significantly enriched pathways (*p* < .05). Eleven of 90 significantly enriched pathways were associated with inflammations such as cytokine–cytokine receptor interaction, NF‐κB signaling pathway, PI3K‐Akt signaling pathway, and TNF signaling pathway (Figure 3a). KEGG enrichment analysis of DEGs between the propionate+IL‐1β group and IL‐1β groups revealed 93 significantly enriched pathways (*p* < .05). Thirteen of 93 significantly enriched pathways were associated with inflammations, such as cytokine–cytokine receptor interaction, PI3K‐Akt signaling pathway, JAK‐STAT signaling pathway, MAPK signaling pathway, NF‐κB signaling pathway, and TNF signaling pathway (Figure 3b). KEGG enrichment analysis of DEGs between the butyrate+IL‐1β and IL‐1β groups revealed 101 significantly enriched pathways (*p* < .05). Fourteen of 101 significantly enriched pathways were associated with inflammations, including cytokine–cytokine receptor interaction, PI3K‐Akt signaling pathway, NF‐κB signaling pathway, MAPK signaling pathway, JAK‐STAT signaling pathway, and Wnt signaling pathway (Figure 3c).

Based on the significance of KEGG pathway enrichment and the number of enriched DEGs, PI3K‐Akt, JAK‐STAT, NF‐κB, and MAPK pathways were selected for validation in vitro. These four pathways were common in the three comparison groups, although JAK‐STAT and MAPK signaling pathways were not significantly enriched in the acetate+IL‐1β group compared with IL‐1β group. The DEGs associated with inflammation from the four key pathways are shown in Table 1.

**TABLE 1:** Key pathways and the major DEGs in these pathways related to inflammation

Pathway	Key DEGs from the pathway		
	IL vs. AIL	IL vs. PIL	IL vs. BIL
PI3K‐Akt signaling pathway	TLR2, NFKB1, PI3KCG, F2R	TLR2, SYK, JAK1, PIK3CG, PIK3CA_B_D, AKT, NFKB1, RAF1	TLR2, SYK, JAK1, PIK3CG, PIK3CA_B_D, AKT, ERK, NFKB1, RAF1,
JAK‐STAT signaling pathway	STAT1, IL−2, SOCS1, AOX	JAK1, STAT1, AKT, SOCS1, AOX, IL−2, RAF1	IL−2, JAK1, STAT1, AKT, AOX, SOCS1, RAF1
NF‐κB signaling pathway	IL−1B, IL−1R1, CD14, VCAM1, NFKB1, IRAK1, CCL21	IL−1R1, IL−1B, SYK, CD14, TLR4, NFKB1, CCL4, CXCL2, CCL21, COX2(PTGS2), IRAK1	IL−1R1, IL−1B, CD14, TLR4, SYK, NFKB1, COX2, CCL4, CXCL2, CCL21, IRAK1
MAPK signaling pathway	NFKB1, IL−1R1, CD14, MAP3K6, IRAK1	NFKB1, JNK, AKT, IL−1A, IL−1R1, IRAK1, CD14, RAF1, MAP3K6, MAP3K5, MAP2K7	CD14, IL−1A, IL−1R1, IRAK1, HRAS, RAF1, ERK, NFKB1, AKT, MAP3K6, MAP3K5, MAP3K1, MAP2K7, JNK

### 3.4 Validation of DGEs with qRT‐PCR

**FIGURE 4:**
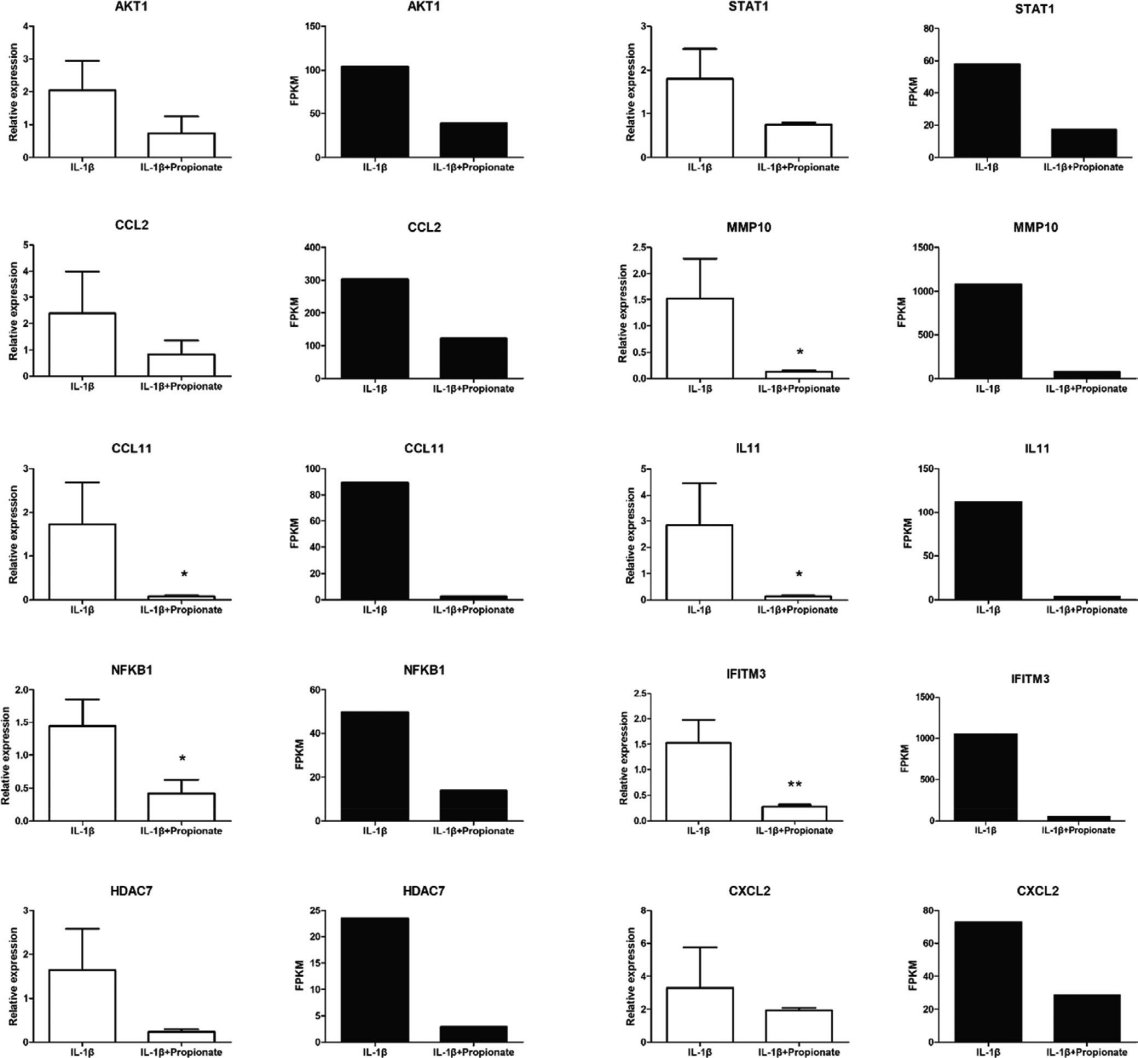
qRT‐PCR results (white) for ten DEGs compared with RNA‐seq results (black). ^∗^ *p* < .05, ^∗∗^ *p* < .01 compared with the IL‐1β group

In order to verify the reliability of RNA‐seq result, we examined the mRNA expression of 10 DEGs related to inflammation between the propionate+IL‐1β and IL‐1β groups by qRT‐PCR. These ten DEGs include thymoma viral proto‐oncogene 1 (AKT1), chemokine (C‐C motif) ligand 2 (CCL2), chemokine (C‐C motif) ligand 11 (CCL11), nuclear factor kappa B subunit 1 (NFKB1), histone deacetylase 7 (HDAC7), signal transducer and activator of transcription 1 (SATA1), matrix metallopeptidase 10 (MMP10), interleukin 11 (IL‐11), interferon‐induced transmembrane protein 3 (IFITM3), and chemokine (C‐X‐C motif) ligand 2 (CXCL2). The results showed that the mRNA levels of 10 genes in the propionate+IL‐1β group were lower than those in the IL‐1β group. Similar trends in qRT‐PCR and RNA‐Seq indicated that the transcriptome sequencing data are reliable (Figure 4).

### 3.5 SCFAs decreased IL‐8 and IL‐6 secretions in IL‐1β‐induced FHs 74 Int cells in vitro

**FIGURE 5:**
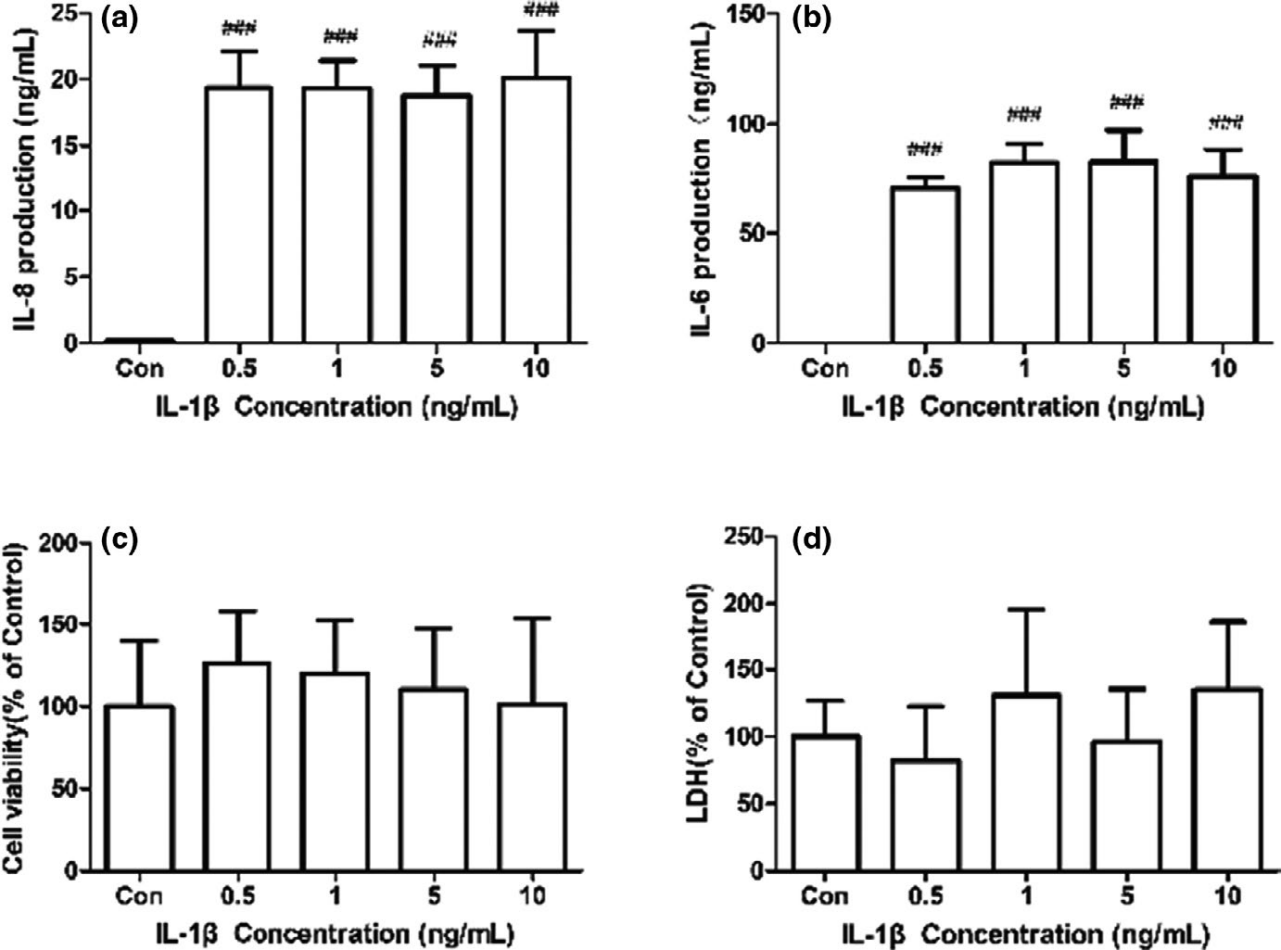
Sensitivity of IL‐8 and IL‐6 expression levels to IL‐1β in FHs 74 Int cells. FHs 74 Int cells were stimulated with or without IL‐1β (0.5–10 ng/ml) for 24 h. IL‐8 (a) and IL‐6 (b) concentrations in FHs 74 Int cells culture supernatant were measured by ELISA kit. Sensitivity of cell viability (c) and cytotoxicity (d) to IL‐1β in cells was measured by CCK‐8 and LDH assay. Data are represented as mean ± *SD*. ^###^ *p* < .001 vs. the control group

To investigate the potential mechanism of the anti‐inflammatory actions of SCFAs, we also conducted investigations in vitro to validate the results of experiments ex vivo. We treated FHs 74 Int cells with IL‐1β to construct FHs 74 Int cells model of inflammation. Four IL‐1β concentrations of 0.5–10 ng/ml increased IL‐8 and IL‐6 concentrations significantly (Figure 5a,b, *p* < .05), indicating that the FHs 74 Int cell model of inflammation was successful. Moreover, there was no significant difference between IL‐8 and IL‐6 productions induced by four different IL‐1β concentrations. As we can see, different concentrations of IL‐1β were not significantly toxic and did not have significant effects on the viability of FHs 74 Int cells (Figure 5c,d). Based on the above results, 0.5 ng/ml was chosen as IL‐1β working concentration in cells.

**FIGURE 6:**
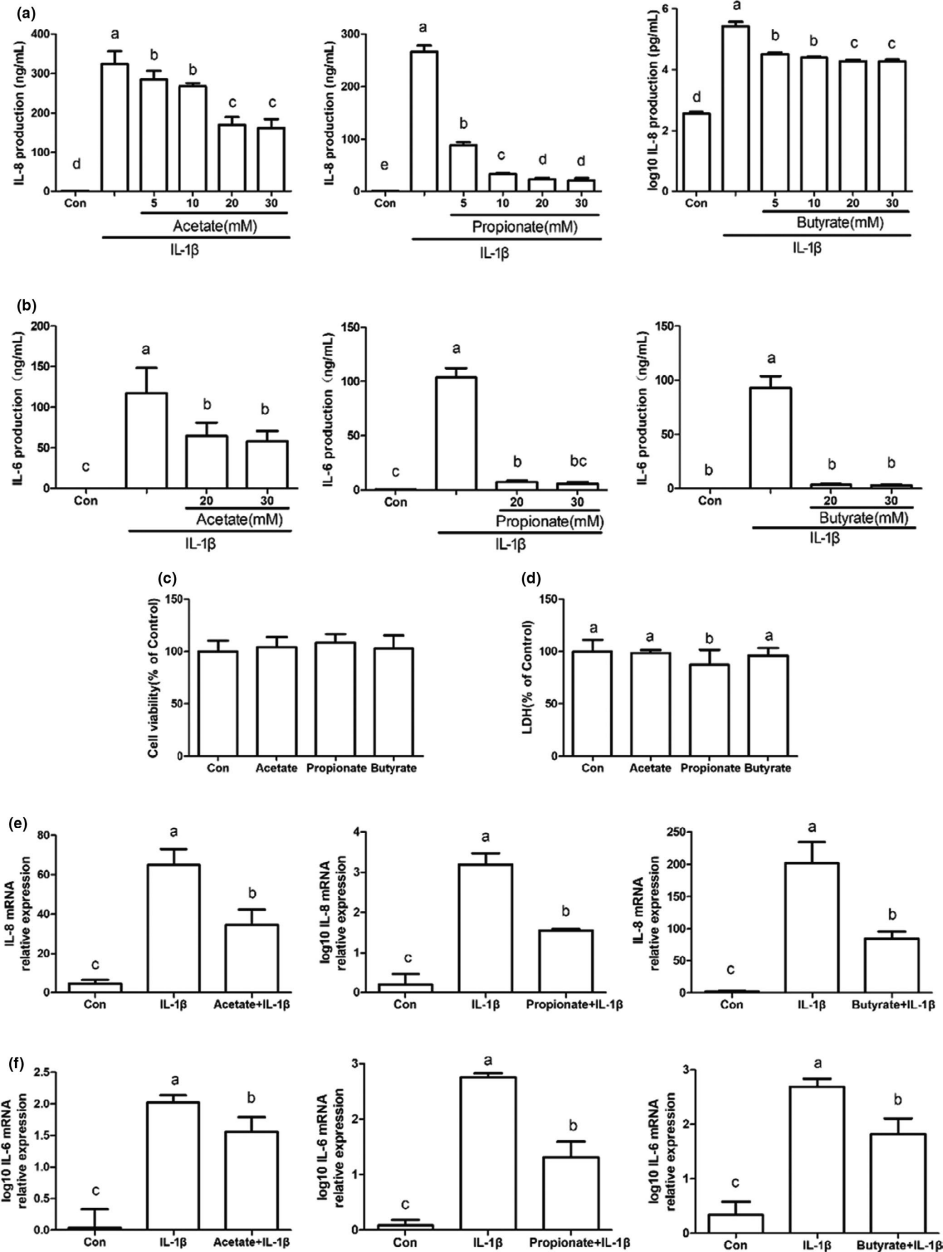
Decrease in IL‐1β‐induced expression levels of IL‐8 and IL‐6 in FHs 74 Int cells by SCFAs. FHs 74 Int cells were pretreated with or without acetate, propionate, or butyrate (a, 5−30 mM, b, 20–30 mM, c–f, 20 mM) for 1 h, and then incubated for 24 h with IL‐1β (0.5 ng/ml). IL‐8 (a) and IL‐6 (b) concentrations in cell cultures supernatant were determined by ELISA kit Sensitivity of cell viability (c) and cytotoxicity (d) to SCFAs was detected by CCK‐8 and LDH assay. IL‐8 mRNA (e) and IL‐6 mRNA (f) levels were evaluated by qRT‐PCR. Data are represented as mean ± *SD*. Data with different letters indicated significant differences (*p* < .05) between groups

IL‐1β‐induced production of IL‐8 was dose dependently suppressed by SCFAs at 5–30 mM (Figure 6a, *p* < .05). SCFAs at 20 and 30 mM also suppressed IL‐6 production induced by IL‐1β (Figure 6b, *p* < .05). Otherwise, the difference in IL‐8 or IL‐6 protein content between the treatments of 20 and 30mM SCFAs was insignificant (Figure 6a,b, *p* > .05). As seen in Figure 6c,d, SCFAs at 20 mM had no negative effect on cells and the three SCFAs are all set at 20 mM.

IL‐8 and IL‐6 mRNA levels in FHs 74 Int cells induced by IL‐1β were verified by qRT‐PCR. As shown in Figure 6e,f, SCFAs significantly reduced IL‐8 and IL‐6 mRNA levels (*p* < .05), which was consistent with ELISA results.

### 3.6 SCFAs suppressed NF‐κB p65, JNK1/2, and ERK1/2 phosphorylation levels in IL‐1β‐induced FHs 74 Int cells in vitro

**FIGURE 7:**
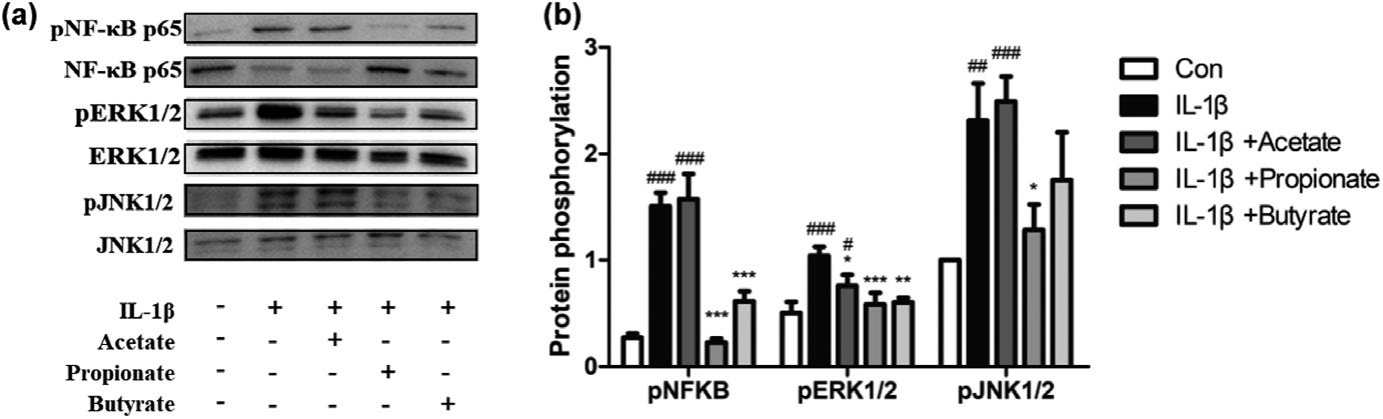
Effect of SCFAs on phosphorylation of NF‐κB p65, JNK1/2, and ERK1/2 in IL‐1β‐induced FHs 74 Int cells. FHs 74 Int cells were pretreated with or without acetate (a, 20 mM), propionate (b, 20 mM), or butyrate (c, 20 mM) for 1 h, and then induced by IL‐1β (0.5 ng/ml) for 24 h. The protein bands were detected by Western blot (a) and the band densities were quantified (b). Data are represented as mean ± *SD*. ^∗^ *p* < .05, ^∗∗^ *p* < .01, ^∗∗∗^ *p* < .001 vs. the IL‐1β group. ^#^ *p* < .05, ^##^ *p* < .01, ^###^ *p* < .001 vs. the control group

Based on KEGG enrichment analysis results (Figure 3 and Table 1), we validated the phosphorylation levels of AKT, JAK1, STAT1, NF‐κB p65, JNK1/2, and ERK 1/2 in IL‐1β‐induced FHs 74 Int cells via Western blot. Figure 7 shows that IL‐1β significantly upregulated NF‐κB p65, JNK1/2, and ERK 1/2 phosphorylation levels compared with the control group (*p* < .05). However, the phosphorylation levels of AKT, JAK1, and STAT1 did not change significantly (data not shown). In IL‐1β‐induced FHs 74 Int cells, acetate only inhibited ERK1/2 phosphorylation (*p* < .05); propionate significantly inhibited the phosphorylation levels of NF‐κB p65, JNK1/2 and ERK 1/2 (*p* < .05); butyrate significantly suppressed NF‐κB p65 and ERK1/2 phosphorylation levels (*p* < .05) but the inhibition of JNK1/2 phosphorylation was not statistically significant (*p* > .05).

### 3.7 The inhibitors of NF‐κB p65, JNK1/2, and ERK 1/2 decreased IL‐8 and IL‐6 mRNA levels in IL‐1β‐induced FHs 74 Int cells in vitro

**FIGURE 8:**
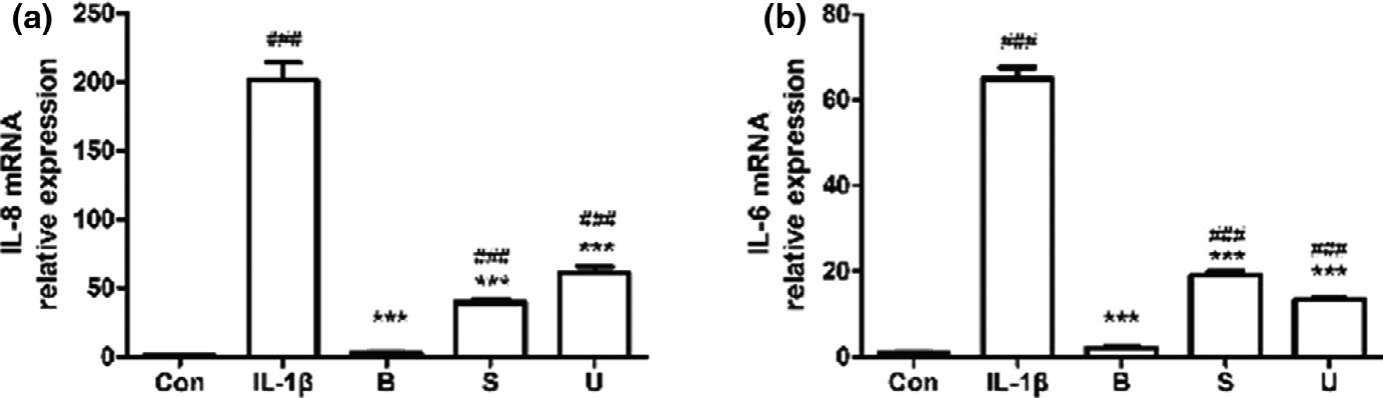
Inhibitors of NF‐κB p65, JNK1/2, and ERK1/2 decreased IL‐1β‐induced IL‐8 (a) and IL‐6 (b) mRNA levels in FHs 74 Int cells. FHs 74 Int cells were pretreated with or without NF‐κB p65 inhibitor BAY 11–7085 (B, 10 μM), JNK1/2 inhibitor SP600125 (S, 20 μM) or ERK1/2 inhibitor U0126 (U, 20 μM) for 1 h, and then induced by IL‐1β for 24 h. The detection of IL‐8 and IL‐6 mRNA levels in FHs 74 Int cells was performed by qRT‐PCR. Data are represented as mean ± *SD*. ^∗∗∗^ *p* < .001 vs. the IL‐1β group. ^###^ *p* < .001 vs. the control group

In order to further confirm that SCFAs regulated inflammatory response induced by IL‐1β through restraining the phosphorylation of NF‐κB p65, JNK1/2, and ERK 1/2, the inhibitors of NF‐κB p65 (BAY 11–7085, 10 μM), JNK1/2 (SP600125, 10 μM), and ERK1/2 (U0126, 20 μM) were used. The results showed that the three inhibitors significantly downregulated the mRNA levels of IL‐8 and IL‐6 in IL‐1β‐induced FHs 74 Int cells (Figure 8, *p* < .05).

## 4 DISCUSSION

Necrotizing enterocolitis is a common intestinal disease in neonates, especially in premature infants. However, the pathogenesis of NEC has not been entirely determined. It has been reported that the inappropriate reaction between colonized bacteria and immature intestine can lead to NEC (Nanthakumar et al., 2011). Intestinal epithelial cells (IECs) are the first defense line for the innate immune system, which maintains intestinal homeostasis (Kinnebrew & Pamer, 2012; Takeuchi & Akira, 2010). Immaturity of IECs and imbalance of the microbiota probably result in intestinal inflammation in preterm infants. Both immune cells and IECs secrete IL‐1β, a proinflammatory cytokine promoting the development of NEC (Haver et al., 2009; Meng et al., 2016). Therefore, we analyzed the roles of SCFAs on IL‐1β‐induced inflammation models of fetal mouse jejunum and FHs 74 Int cells and then explored the mechanisms. The results showed that SCFAs mediated anti‐inflammatory actions in the fetal mouse jejunum and FHs 74 Int cell inflammation models by reducing NF‐κB p65, JNK1/2, and ERK1/2 phosphorylation levels.

In this study, IL‐1β significantly activated CXCL2 (an IL‐8 homologue) production in fetal mouse jejunum cultures and IL‐8 and IL‐6 productions in FHs 74 Int cells. One of the characteristics of intestinal inflammation is neutrophil recruitment from blood to the sites of inflammatory (Hung & Suzuki, 2018). There is growing evidence that IL‐8 and IL‐6 secreted by intestinal epithelial cells have essential effects on neutrophil recruitment (Hung & Suzuki, 2018). Therefore, the inhibition of IL‐8 and IL‐6 production may be an effective way for preventing and reducing inflammation (Holcombe et al., 1994; Jones et al., 2011). It is known that SCFAs have anti‐inflammatory actions on the mature intestine, such as mouse colon tissues and human colon epithelial Caco‐2 cells (Hung & Suzuki, 2018; Kim et al., 2018; Singh et al., 2014). However, there are few studies about the actions of SCFAs on immature intestinal inflammation. To our knowledge, only one study showed acetate, propionate, and butyrate had anti‐inflammatory actions on fetal mouse intestine and fetal small intestinal epithelial H4 cell line stimulated by IL‐1β (Zheng et al., 2020). In this study, all three SCFAs significantly reduced IL‐1β‐induced productions of IL‐8 and IL‐6 or CXCL2 in both models, which was consistent with the result of the study by Zheng et al. (2020). In addition, differences in the anti‐inflammatory actions of the three SCFAs at the same concentration on FHs 74 Int cells were observed, and the order of potency of SCFAs inhibiting IL‐8 and IL‐6 productions induced by IL‐1β was acetate < propionate < butyrate. Our observations were consistent with the study of Hung and Suzuki; this order was obviously related to the physiological concentrations of SCFAs in intestine (Hung & Suzuki, 2018).

The anti‐inflammatory effect of SCFAs was also reported in vivo. The weaned piglets treated with a mixture of SCFAs showed that the relative mRNA expression of IL‐1β in the ileum and IL‐1β and IL‐8 in the colon was significantly decreased by treating with low‐dose SCFAs (acetate, propionate, and butyrate; 20.04, 7.71, and 4.89 mM, respectively), whereas the relative mRNA expression of IL‐1β and IL‐8 in the jejunum, ileum, and colon was significantly decreased by treating with high‐dose SCFAs (acetate, propionate, and butyrate; 40.08, 15.41, and 9.78 mM respectively); overall, the anti‐inflammatory effect of high‐dose SCFAs was more significant (Diao et al., 2019). Another study showed the anti‐inflammatory effect of low‐dose SCFAs (3 mM) on a TNBS‐induced zebrafish larval intestinal inflammation model (Fénero et al., 2021). This demonstrates that the anti‐inflammatory effect of the mixed SCFAs may depend on organ differences, species differences, and administration methods.

The combination of chemicals may perform different interaction effects, such as synergistic, antagonistic, or additive effects (Ficheux et al., 2012). Asarat (2015) observed that the individual 20 mM acetate, propionate, butyrate, and their mixed SCFAs had no significant difference in inhibiting the production of IL‐8 in LPS‐induced normal intestinal epithelial (T4056) or adenocarcinoma‐derived (HT‐29) cell lines (Asarat et al., 2015). It could be speculated that there was no synergistic anti‐inflammatory effect among these three SCFAs. However, the interaction cannot be evaluated simply by phenotype analysis. It has been reported that the methods for evaluating interactions mainly include the comparison of the measured values with the theoretical expected values (Huang et al., 2019), full‐factorial design (Zheng et al., 2017), and isobologram (Gao et al., 2016). At present, there is still a lack of research into the anti‐inflammatory interactions of acetate, propionate, and butyrate. Therefore, we will further investigate the anti‐inflammatory interaction of SCFAs mixture and whether low dose of mixed SCFAs has significant anti‐inflammatory effect based on this study.

We explored the anti‐inflammatory mechanisms of the three SCFAs on the fetal mouse small intestine through RNA‐seq. According to the results of KEGG pathway enrichment analysis, we verified the phosphorylation levels of AKT, JAK1, STAT1, NF‐κB p65, JNK1/2, and ERK1/2 in FHs 74 Int cells via Western blot, and then, an inhibition assay was carried out. The results showed that SCFAs suppressed IL‐8 and IL‐6 expression induced by IL‐1β through inhibition of NF‐κB p65, JNK1/2, and ERK1/2 phosphorylation in FHs 74 Int cells. According to the literature, the activation of NF‐κB, JNK, and ERK, which play essential roles in IL‐1β‐induced inflammation (Choi & Lee, 2010; Csaki et al., 2009; Greten et al., 2007), promoted inflammatory cytokine expression in intestinal cells (Hoffmann et al., 2002). NF‐κB, a key transcription factor regulating cell differentiation, inflammatory response, and immune response, is separated from Iκ B and translocated to the nucleus after stimulation by proinflammatory cytokines (Jiang et al., 2017). The inducers of NF‐κB consist of lipopolysaccharide, IL‐1, and TNF‐α (Herfarth et al., 2000). Previous studies have reported that butyrate had an anti‐inflammatory action in colon adenocarcinoma through inhibiting NF‐κB pathway (Chen et al., 2018; Lhrs et al., 2001). Also, Hung and Suzuki showed that acetate, propionate, and butyrate suppressed the activation of NF‐κB induced by TNF‐α, although the inhibitory effects of acetate and butyrate on NF‐κB phosphorylation were statistically insignificant (Hung & Suzuki, 2018). In this study, propionate and butyrate significantly suppressed IL‐1β‐stimulated phosphorylation of NF‐κB p65. Moreover, ERK and JNK belong to MAPK signaling pathway, which plays an essential role in a number of host reactions and is one of the primary pathways that transmit signals to immediate early genes involved in cytokine response regulation (Hammaker et al., 2003). Huang and Suzuki showed that acetate, propionate, and butyrate significantly suppressed the phosphorylation of JNK1/2 and ERK1/2 in TNF‐α‐induced Caco‐2 cells (Hung & Suzuki, 2018), and Kobayashi et al. showed that acetate, propionate, and butyrate downregulated the phosphorylation of JNK in TNF‐α‐induced human renal cortical epithelial cells (HRCEs) (Kobayashi et al., 2017). In our study, the three SCFAs also significantly inhibited IL‐1β‐induced phosphorylation of ERK1/2 in FHs 74 Int cells, which was in agreement with the results from the study of Hung and Suzuki (Hung & Suzuki, 2018), but only propionate significantly inhibited the phosphorylation of JNK1/2. The results of previous studies indicate that the effect of SCFAs on signaling pathway was affected by different cells or drugs inducing inflammatory. On the contrary, it was possible that the concentrations of SCFAs in this experiment were not sufficient to significantly suppress the phosphorylation level of the pathway. Interestingly, the phosphorylation levels of JNK1/2 and NF‐κB p65 showed a slight increase after acetate treatment. Kasperczyk et al. showed that betulinic acid (BA) can activate NF‐κB in SH‐EP neuroblastoma cells at a dose of 5–10 μg/ml, but not at 0–5 μg/ml (Kasperczyk et al., 2005). The evidence demonstrates that the concentration of drugs had a great influence on the signaling pathway. Finally, we observed that the inhibitors of NF‐κB p65, JNK1/2, and ERK1/2 suppressed IL‐1β‐induced productions of IL‐8 and IL‐6 significantly, which showed similar effects to SCFAs. Therefore, based on these results, we indicated that the three SCFAs play anti‐inflammatory roles by inhibiting the phosphorylation of these pathways.

The anti‐inflammatory actions of SCFAs have also been reported to be associated with their inhibition of HDAC (Chang et al., 2014; Koh et al., 2016; Li et al., 2017). For example, butyrate suppressed colonic inflammation through acting as an HDAC inhibitor (Zimmerman et al., 2012). Through RNA‐seq analysis, we found HDAC genes were downregulated when IL‐1β‐induced fetal mouse jejunum cultures were stimulated by SCFAs (Table S2). And then, it was verified by qRT‐PCR that butyrate significantly inhibited HDAC7 expression induced by IL‐1β ex vivo. In vitro, propionate and butyrate but not acetate inhibited HDAC5 mRNA level in FHs 74 Int cells induced by IL‐1β (Figure S1a, *p* < .05). Furthermore, we also found inhibitors of HDAC5 decreased the mRNA levels of IL‐8 and IL‐6 significantly (Figure S1, *p* < .05). Based on these results, we preliminarily believe that SCFAs protect against immature small intestinal inflammation at least in part by inhibiting HDAC, which needs further study.

## 5 CONCLUSION

In conclusion, acetate, propionate, and butyrate had a protective effect on fetal ICR mouse jejunum culture inflammation and human fetal small intestinal epithelial FHs Int 74 cell inflammation through inhibiting phosphorylation of NF‐κB p65, JNK1/2, and ERK1/2 pathways, as well as inhibiting HDAC. Therefore, SCFAs may be a new drug for the prevention of NEC.

## CONFLICT OF INTEREST

The authors have no conflict of interest to declare.

## AUTHOR CONTRIBUTIONS

**Shengnan Huang:** dataCuration (equal); formalAnalysis (equal); investigation (lead); methodology (equal); visualization (equal); writingOriginalDraft (lead); writingReviewEditing (equal). **Yanan Gao:** conceptualization (equal); dataCuration (lead); methodology (equal); supervision (equal); visualization (equal); writingReviewEditing (lead). **Ziwei Wang:** dataCuration (equal); formalAnalysis (equal); investigation (equal); software (equal); visualization (equal). **Xue Yang:** investigation (equal); methodology (equal); visualization (equal). **Jiaqi Wang:** conceptualization (equal); fundingAcquisition (equal); resources (lead). **Nan Zheng:** conceptualization (lead); dataCuration (equal); fundingAcquisition (lead); projectAdministration (lead); resources (equal); supervision (equal).

## ETHICAL APPROVAL

These animal experiments were approved by Animal Experimental Ethics Committee of Institute of Animal Sciences, Chinese Academy of Agricultural Science (IAS2019‐65).

## Supporting information

App S1

## Data Availability

The data that support the findings of this study are available from the corresponding author upon reasonable request.
