# Supplementary material for: Anti‐inflammatory actions of acetate, propionate, and butyrate in fetal mouse jejunum cultures ex vivo and immature small intestinal cells in vitro
Source: Food Sci Nutr. 2022 Jan 18;10(2):564–76. doi: 10.1002/fsn3.2682 (PMC8825721; doi:10.1002/fsn3.2682)
Supplement: Supplementary file 1 — App S1 [file FSN3-10-564-s001.docx]

**Appendix S1**


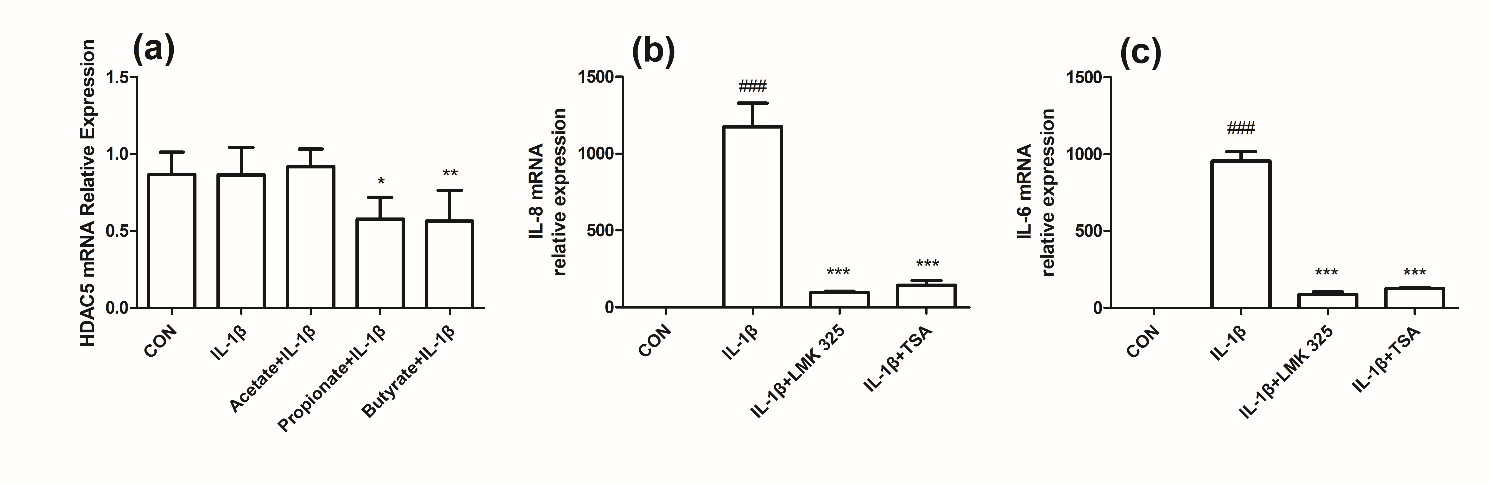


**FIGURE S1**. Effect of HDAC5 on inflammation of FHs 74 Int cells. Propionate and butyrate inhibited HDAC5 mRNA levels in IL-1β-induced FHs 74 Int cells(a). FHs 74 Int cells were pretreated with acetate (20 mM), propionate (20 mM), or butyrate (20 mM) for 1 h in the absence or presence, and then stimulated with IL-1β (0.5 ng/mL) for 24 h. LMK 325 and TSA decreased IL-1β-induced IL-8 (b) and IL-6 (c) mRNA levels in FHs 74 Int cells. FHs 74 Int cells were pretreated with LMK 325 (5 mM) or TSA (1 µM) for 1 h in the absence or presence, and then stimulated with IL-1β for 24 h. HDAC5, IL-8, and IL-6 mRNA levels in cells were measured by qRT-PCR. Data are represented as the mean ± *SD*. ^∗^ *p* < .05, ^∗∗^ *p* < .01, ^∗∗∗^ *p* < .001 vs. IL-1β group. ^###^ p < .001 vs. Control group.

###### **TABLE S1.** Primer sequences for qRT-PCR

| Gene | Forward Sequence (5‘-3’) | Reverse Sequence (5‘-3’) | References |
| --- | --- | --- | --- |
| Human IL-8 | ATGACTTCCAAGCTGGCCGTGGCT | TCTCAGCCCTCTTCAAAAACTTCTC | Hung & Suzuki, 2018 |
| Human IL-6 | CCTTCCAAAGATGGCTGAAA | CAGGGGTGGTTATTGCATCT | Hung & Suzuki, 2018 |
| Human β-actin | TTTTAGGATGGCAAGGGACTT | GATGAGATTGGCATGGCTTTA | Hung & Suzuki, 2018 |
| Mouse β-actin | AGGTCATCACTATTGGCAACG | ATCTCCTTCTGCATCCTGTCA | Hung & Suzuki, 2018 |
| AKT1 | CCCTTCTACAACCAGGACCA | ATACACATCCTGCCACACGA | this study |
| CCL2 | AGGTCCCTGTCATGCTTCTG | TCTGGACCCATTCCTTCTTG | this study |
| CCL11 | TCCACAGCGCTTCTATTCCT | CTATGGCTTTCAGGGTGCAT | this study |
| NFKB1 | CTGACCTGAGCCTTCTGGAC | GCAGGCTATTGCTCATCACA | this study |
| HDAC7 | TGAAGAATGGCTTTGCTGTG | CACTGGGGTCCTGGTAGAAA | this study |
| STAT1 | TGGTGAAATTGCAAGAGCTG | CAGACTTCCGTTGGTGGATT | this study |
| MMP10 | CAGGTGTGGTGTTCCTGATG | GGAGAAAGTGAGTGGGGTCA | this study |
| IL11 | TGACGGAGATCACAGTCTGG | GAGCTGTAAACGGCGGAGTA | this study |
| VIM | ATGCTTCTCTGGCACGTCTT | AGCCACGCTTTCATACTGCT | this study |
| CXCL2 | AGTGAACTGCGCTGTCAATG | TTCAGGGTCAAGGCAAACTT | this study |

###### **TABLE S2.** Effect of acetate, propionate, and butyrate on HDAC genes in inflammatory model of jejunum organ cultures

| Gene | IL vs. AIL | | IL vs. PIL | | IL vs. BIL | |
| --- | --- | --- | --- | --- | --- | --- |
|  | log_2_(fc) | trend | log_2_(fc) | trend | log_2_(fc) | trend |
| HDAC7 | / | / | -3.003074 | DOWN | -3.481297 | DOWN |
| HDAC8 | / | / | / | / | -1.01016 | DOWN |
| HDAC9 | -1.272253 | DOWN | -2.637997 | DOWN | -2.67013 | DOWN |
